# Discovery of a Natural Product-Like c-*myc* G-Quadruplex DNA Groove-Binder by Molecular Docking

**DOI:** 10.1371/journal.pone.0043278

**Published:** 2012-08-17

**Authors:** Dik-Lung Ma, Daniel Shiu-Hin Chan, Wai-Chung Fu, Hong-Zhang He, Hui Yang, Siu-Cheong Yan, Chung-Hang Leung

**Affiliations:** 1 Department of Chemistry, Hong Kong Baptist University, Kowloon Tong, Hong Kong, China; 2 Department of Applied Biology & Chemical Technology, The Hong Kong Polytechnic University, Hong Kong, China; 3 Institute of Chinese Medical Sciences, University of Macau, Macao SAR, China; 4 State Key Laboratory of Quality Research in Chinese Medicine, University of Macau, Macao SAR, China; University of Quebect at Trois-Rivieres, Canada

## Abstract

The natural product-like carbamide (**1**) has been identified as a stabilizer of the c-*myc* G-quadruplex through high-throughput virtual screening. NMR and molecular modeling experiments revealed a groove-binding mode for **1**. The biological activity of **1** against the c-*myc* G-quadruplex was confirmed by its ability to inhibit *Taq* polymerase-mediated DNA extension and c-*myc* expression *in vitro*, demonstrating that **1** is able to control c-*myc* gene expression at the transcriptional level presumably through the stabilization of the c-*myc* promoter G-quadruplex. Furthermore, the interaction between carbamide analogues and the c-*myc* G-quadruplex was also investigated by *in vitro* experiments in order to generate a brief structure-activity relationship (SAR) for the observed potency of carbamide **1**.

## Introduction

G-quadruplexes are non-canonical DNA structures comprised of planar arrangements of guanine tetrads stabilized by Hoogsteen hydrogen bonding and monovalent cations. Guanine-rich G-quadruplex-forming sequences appear frequently in telomeres and in the promoter regions of growth control genes such as c-*myc* [1]. Stabilizing the G-quadruplex structure with small molecule ligands in order to inhibit telomerase activity or repress oncogene expression has thus emerged as a potential strategy for the treatment of cancer [2]. The design of G-quadruplex ligands has traditionally utilized planar aromatic scaffolds that form strong π–π stacking interactions with the 5′ or 3′-terminal guanine tetrad of the quadruplex core [3]. However, targeting the grooves and loops of the G-quadruplex can potentially offer a higher degree of selectivity between the different quadruplex topologies due to the higher structural heterogeneity in the external regions of the G-quadruplex [4].

The c-*myc* oncogene encodes a transcription factor that controls important elements involved in cell cycle regulation, cell growth and proliferation, and apoptosis. This gene is believed to regulate 15% of all gene expression, and the overexpression of c-*myc* has been associated with the progression of malignant tumors [5]. The nuclear hypersensitivity element III_1_ (NHE III_1_) is a guanine-rich sequence located upstream of the c-*myc* P1 promoter that controls 80–90% of c-*myc* transcription [6]. Pu27, a 27-nucleotide (nt) six-guanine-tract sequence residing within the NHE III_1_, has been shown to fold into multiple G-quadruplex structures in solution, including those resembling the so-called “propeller-type” parallel intramolecular G-quadruplex [7]. Small molecule ligands that have been reported to stabilize the c-*myc* NHE III_1_ G-quadruplex(es) and inhibit c-*myc* oncogene transcription include, but are not limited to, cationic porphyrins [8], quindoline derivatives [9] and platinum complexes [10]. We have previously identified a c-*myc* G-quadruplex stabilizing natural product Fonsecin B, which was predicted to bind to the c-*myc* G-quadruplex through end-stacking at the 3′-terminus via its extended aromatic interface, by high throughput virtual screening [11].

Surprisingly, however, only a few G-quadruplex groove-binders have been reported to date. Shafer *et al.* suggested that 3,3′-diethyloxadicarbocyanine (DODC) is able to bind to the groove region of a dimeric G-quadruplex [12], which was subsequently validated in a later study [13], [14]. In addition, Wilson and co-workers have demonstrated that certain heterocyclic diamidines are able to target the groove regions of the human telomeric G-quadruplex [14]. Meanwhile, Randazzo and co-workers have used virtual screening and NMR experiments to identify groove-binding ligands targeting the [d(TGGGGT)]_4_ G-quadruplex from a database of 6,000 commercial compounds [15]. Encouraged by these ideas, we were interested to see if we could apply our molecular modeling methods to identify new groove-binding scaffolds targeting the c-*myc* G-quadruplex from a natural product and natural product-like database. Natural products are a rich source of novel chemical scaffolds and their utility in the discovery of new medicines has been extensively documented [16]. The synthetic minor groove-binding dye Hoechst 33258 has been reported to interact with a AAGGT loop (not present in Pu27) in a 31-nt c-*myc* G-quadruplex [17]. The Pu27 parallel intramolecular c-*myc* G-quadruplex contains three propeller loops with an all-anti arrangement of guanines, except for a 3′ snapback *syn* guanine at one of the termini. The unique topology of the looping residues and the snapback loop in the Pu27 c-*myc* G-quadruplex gives rise to specific recognition sites in the quadruplex groove regions which can be exploited by selective small molecule groove-binders [18]. To our knowledge, no other c-*myc* G-quadruplex groove-binder has been reported.

**Figure 1:**
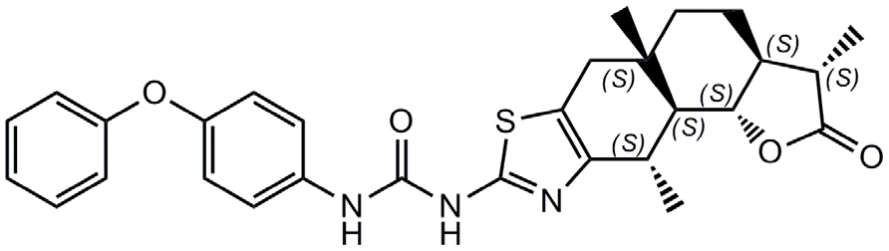
Chemical structure of carbamide 1.

We constructed a model of the intramolecular G-quadruplex loop isomer of NHE III_1_ using the X-ray crystal structure of the intramolecular human telomeric G-quadruplex DNA (PDB code: 1KF1) [7]. This model has previously been utilized to discover c-*myc* G-quadruplex stabilizing ligands derived from natural products [11], quindoline compounds [9] and platinum(II) Schiff-base complexes [10]. In the present investigation, we restricted the search area to include only the groove areas in order to identify novel groove-binding ligands of the c-*myc* G-quadruplex. Over 20,000 compounds from a database of natural product and natural product-like structures were screened *in silico*. The continuously flexible ligands were docked to a grid representation of the receptor and assigned a score reflecting the quality of the complex according to the ICM method [ICM-Pro 3.6-1d molecular docking software (Molsoft)] [19]. The highest-scoring compounds were tested in a preliminary PCR stop assay to assess their ability to stabilize the c-*myc* G-quadruplex, and carbamide **1** emerged as the top candidate (Figure 1).

## Results and Discussion

To our knowledge, no biological activity or any other properties of carbamide **1** have been reported in the literature. Structurally, **1** consists of a diphenyl ether unit and a tetracyclic moiety linked together via an urea functionality. The tetracyclic core contains the sesquiterpene lactone skeleton found in α-santonin, a common constituent of Artemisia species that are widely used in traditional Chinese and Indian herbal remedies for the treatment of inflammation and other conditions [20]. A biology-oriented synthesis (BIOS) library containing **1** and close derivatives were synthesized from α-santonin by Schwarz and co-workers through a series of modifications, including ring fusion of a 2-thiazole moiety followed by reaction with various amides, to yield a series of new carbamides that were active against 5-lipoxygenase, a mediator in allergy and inflammation [21]. On the other hand, Sung and co-workers reported a series of carbamide analogues that can suppress the activity of cyclooxygenase-2 (COX-2) [22]. While these derivatives were all represented in the chemical database used in our study, **1** displayed the highest activity *in vitro*. This suggests that the variable diphenyl ether unit may play a significant role in enhancing the binding of **1** to the G-quadruplex.

**Figure 2:**
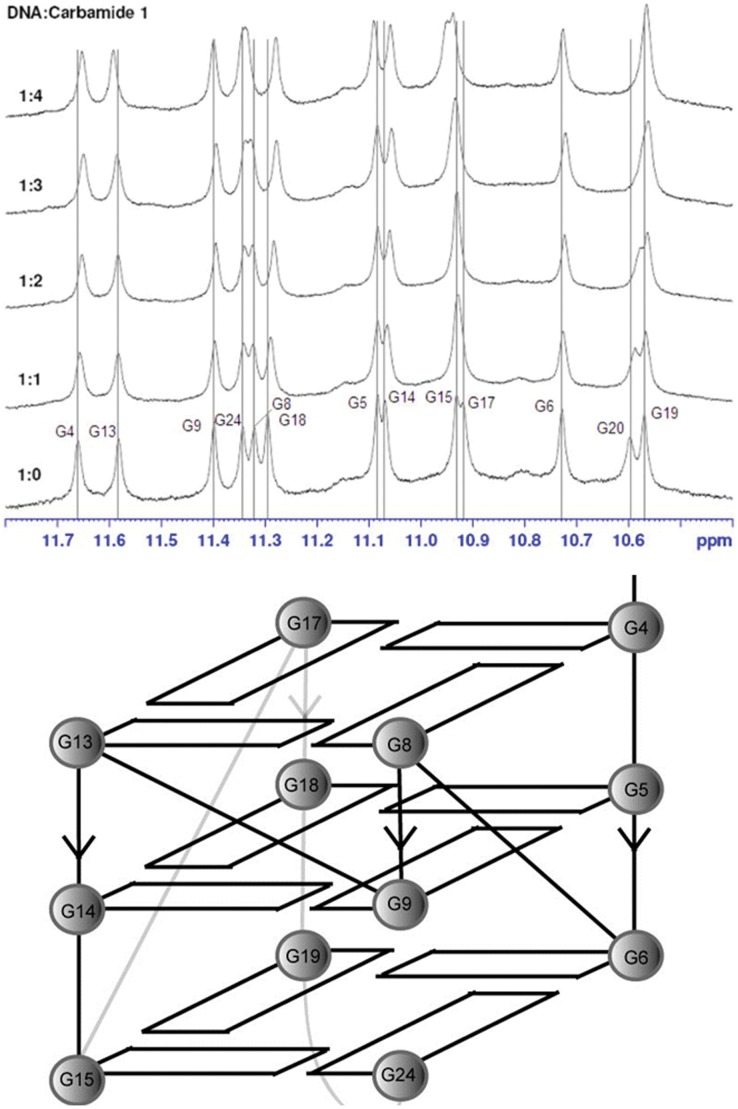
NMR titration of 1 against the c-*myc* G-quadruplex Pu24I. [**1**]/[Pu24I] = 0–4.0. [Pu24I] = 1.0 mM (upper panel). Schematic diagram of the c-*myc* G-quadruplex with the assigned guanines (lower panel).

To verify the mode of binding of compound **1** to the c-*myc* G-quadruplex structure, NMR titration experiments were performed to identify the ligand binding sites. A truncated 24-nt c-*myc* sequence Pu24I containing a guanine-to-inosine substitution was used for this investigation. The triad-containing diagonal loop (G20–A21–A22–G23) of the c-*myc* sequence Pu24I connects G19 to G24 and provides a fold-back configuration for G24 while the double-chain-reversal loops (T7, T16, I10–G11–A12) connect G6 to G8, G15 to G17 and G9 to G13 respectively, thus bridging the G-tetrad layers [23]. Pu24I has been shown [18] to give a higher quality NMR spectrum compared to the full-length sequence Pu27, which forms multiple G-quadruplex topologies in K^+^ solution. The NMR spectrum of Pu24I in the absence of **1** matches that reported previously [18]. We then titrated compound **1** into a solution of Pu24I (1.0 mM) at ratios of [**1**]/[Pu24I]  = 0–4.0. Over the course of the titration, we observed appreciable shifts of 0.02 ppm or higher in the imino proton region for G4, G14, G17, G18 and G20, suggesting that these residues are most involved in ligand binding (Figure 2). These shifts were statistically significant (Figure S1, S2 and Table S1). As these nucleotides are not collectively involved in the formation of a single tetrad at either termini, this suggests that **1** binds Pu24I through external interactions at the grooves or loops of the G-quadruplex. However, partial stacking interactions with the terminal G-quartets cannot be ruled out. Also, since G4, G17 and G18 are located on the opposite side of the G-quadruplex structure compared to G14 and G20, it is likely that two molecules of **1** are bound to Pu24I at the same time. Furthermore, diminishing changes in the chemical shift values of the relevant imino protons were observed as the [**1**]/[Pu24I] ratio was increased from 2.0 to 4.0. This suggests that the binding stoichiometry of **1** to the c-*myc* G-quadruplex is likely to be 2∶1. By comparison, end-stackers such as TMPyP4 have been reported to perturb the guanine residues of the 5′-tetrad (G4, G8, G13 and G17) in the Pu24I NMR spectrum [18].

**Figure 3:**
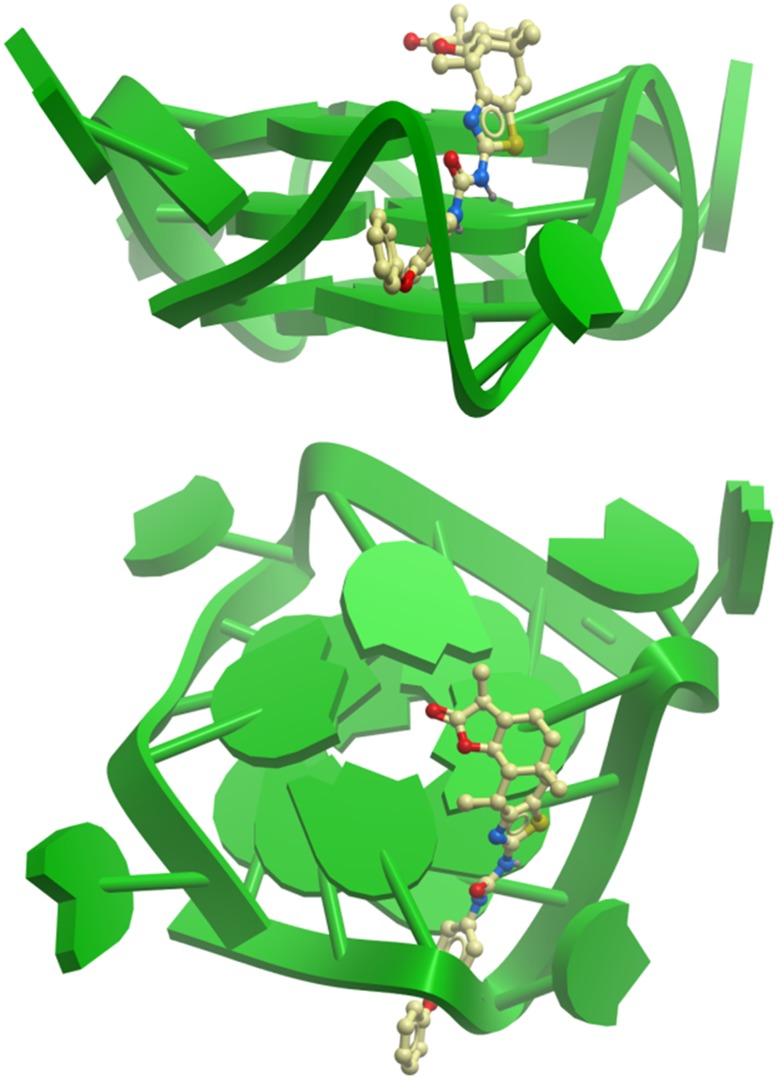
Hypothetical molecular models showing the Side view (upper panel); Top view (lower panel) of the interactions of 1 with the c-*myc* G-quadruplex structure. The G-quadruplex is depicted as a ribbon representation (green), while **1** is depicted as a space-filling representation showing carbon (beige), oxygen (red), nitrogen (blue) and sulfur (yellow) atoms.

To further investigate the mode of binding, we performed molecular modeling of carbamide **1** with the NHE III_1_ intramolecular G-quadruplex loop isomer model. The predominance of the 1∶2:1 loop isomer in the c-*myc* parallel G-quadruplex structure has been previously demonstrated [24]. As neither NMR nor X-ray crystal structure for the c-*myc* NHE III_1_ 1:2∶1 loop isomer was available, we constructed a model from the known, closely related X-ray crystal structure of the human intramolecular telomeric G-quadruplex DNA. We used a truncated 18-nt sequence Pu18 [5′-AGGGTGGGGAGGGTGGGG-3′] since the guanine nucleotides G2–G5 in the c-*myc* sequence [5′-TGGGGAGGGTGGGGAGGGTGGGGAAGG-3′] are not involved in G-quartet formation. Our molecular model of the intramolecular c-*myc* G-quadruplex consists of three G-quartets that are associatively formed from four parallel guanine triads (G2–G4, G6–G8, G11–G13, G16–G18), and three chain-reversal loops (T5, G9–A10, T14–G15). The molecular docking results show that **1** binds strongly to c-*myc* G-quadruplex DNA with a binding energy of –47.66 kcal/mol (Figure 3). In the low energy binding conformation of **1** to Pu18, the molecule is observed to interact extensively with the groove regions of the G-quadruplex, contacting the T14–G15 loop as well as the external groove of G11–G13 (Figure 3, upper panel). The *cis*-B/C fusion of **1** allows the A/B face of the molecule to wrap around the top of the G-quadruplex and form hydrophobic interactions with G11 of the 5′-quartet (Figure 3, lower panel). The snug position of the diphenyl ether unit in the groove of the c-*myc* G-quadruplex may explain the higher activity of **1** compared to related carbamides in the library that bear other functional groups. The molecular modeling calculations are consistent with the NMR results, which show ligand binding to guanine nucleotides from all three G-quartet levels. Although the topologies of Pu18, Pu24I, and the myriad of other G-quadruplexes available in the NHE III_1_ vary to a significant degree, the artificial stabilization of a particular quadruplex conformation could, in principle, still exert a useful pharmacological effect on c-*myc* transcription *in vitro*.

**Figure 4:**
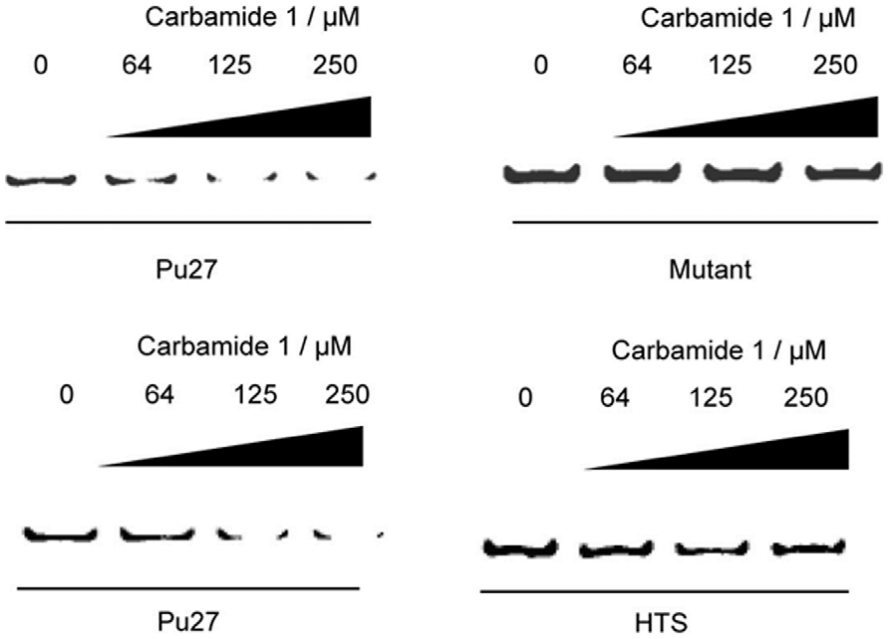
PCR stop assay with c-*myc* Pu27 and its mutant Pu27_mut_ (upper panel) and HTS G-quadruplex (lower panel). A decrease in the PCR amplification product was observed for c-*myc* at 250 µM of **1** but not for its mutant. No significant inhibition of the HTS PCR product was observed.

To validate our NMR and molecular modeling results, we performed a PCR stop assay using the Pu27 c-*myc* G-quadruplex. We observed a decrease in the intensity of the 43 bp PCR product upon addition of **1** at 125 µM indicating that **1** is able to stabilize the c-*myc* G-quadruplex and block DNA amplification by *Taq* polymerase (Figure 4, upper panel). However, **1** had no effect on the amplification of a non-quadruplex-forming c-*myc* mutant sequence. Furthermore, carbamide **1** was selective for the c-*myc* G-quadruplex over the human telomeric G-quadruplex (Figure 4, lower panel). Although the potency of **1** against c-*myc* was modest, this could be explained by the fact that **1** is neutral rather than positively-charged, which reduces the potential coulombic interactions available for binding. Furthermore, groove-binders generally bind G-quadruplexes less avidly compared to end-stackers.

**Figure 5:**
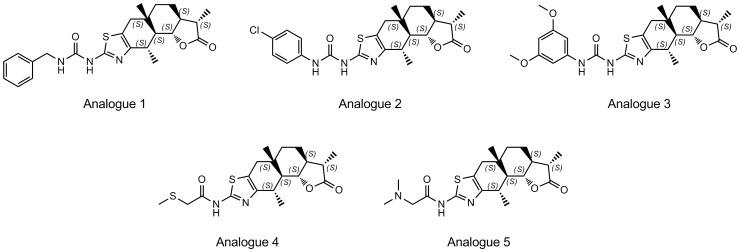
Chemical structures of carbamide 1 analogues (1–5).

**Figure 6:**
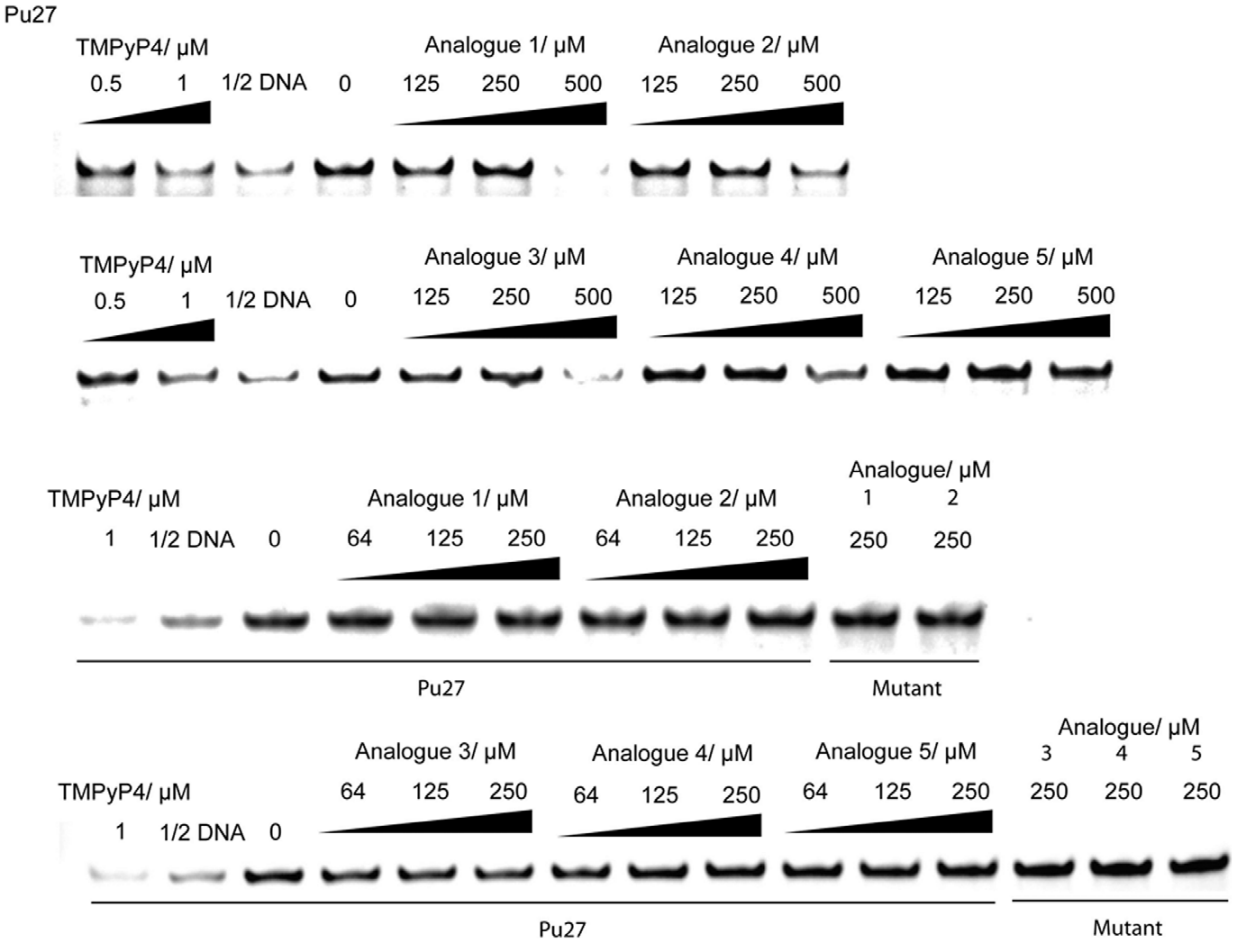
PCR stop assays of the c-*myc* G-quadruplex with increasing concentrations of carbamide 1 analogues.

To investigate the effect of different structural features on the interaction of the ligands against the c-*myc* G-quadruplex and to generate a brief structure-activity relationship (SAR) for carbamide derivatives, we evaluated the c-*myc* G-quadruplex-stabilizing activity of a series of analogues of **1** (Figure 5) in the PCR stop assay. The results showed that inhibition of the PCR product was only observed at higher concentrations (500 µM) of some of the analogues (Figure 6). The modest c-*myc* G-quadruplex stabilizing activities of analogues 1 and 3 could possibly be attributed to the presence of phenyl substituents that partly mimic the diphenyl ether moiety of **1**. On the other hand, the presence of an electronegative chlorine atom on the phenyl group of analogue 2 may have resulted in additional constraints due to enhanced electrostatic repulsion and weakened Van der Waals interaction, resulting in lowered activity. Analogues 4 and 5 that lack aromatic groups attached to the linker were inactive at concentrations of 500 µM. These results further corroborate our molecular modelling analysis which suggested that the diphenyl ether moiety in **1** contributes significantly to quadruplex binding and stabilizing ability.

**Figure 7:**
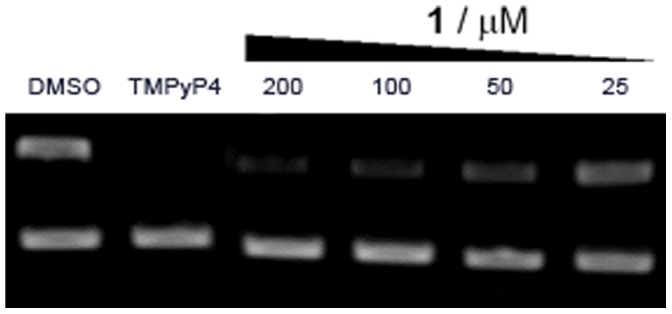
RT-PCR results showing dose-dependent decrease in c-*myc* transcription (upper panel) after incubation of HepG2 cells with 1 (0–200 µM) or TMPyP4 (50 µM) for 16 h. Equal RNA loading was confirmed by β-actin (lower panel).

We next examined whether **1** could inhibit c-*myc* expression in a cellular context. To this end, human hepatocarcinoma (HepG2) cells were incubated with **1** (0–200 µM) for 16 h, and c-*myc* RNA levels were quantified using reverse transcriptase PCR (RT-PCR). Encouragingly, a dose-dependent decrease in c-*myc* RNA concentration was observed, with an estimated IC_50_ value of 50 µM (Figure 7, upper panel). This decrease in c-*myc* RNA concentration was expected to be, at least in part, due to the **1-**mediated stabilization of the c-*myc* NHE III_1_ G-quadruplex structure(s), blocking transcription of c-*myc* by RNA polymerase. The higher potency of **1** in the cellular assay compared to the cell-free assay could be possibly attributed to a concentration of the compound inside the cells or the nucleus of the cells. Alternatively, the transcription machinery inside cells may be more susceptible to the aberrant DNA structures induced by **1** compared to the generally more robust *Taq* polymerase. Lastly, c-*myc* RNA levels could be affected *via* another mechanism besides that of G-quadruplex stabilization.

### Conclusion

In conclusion, we have identified carbamide **1** as a stabilizer of c-*myc* G-quadruplex DNA through structure-based virtual screening, using a unique model of the c-*myc* intramolecular parallel-stranded 1∶2:1 loop isomer constructed from the X-ray crystal structure of the related human telomeric G-quadruplex. The virtual screening campaign was conducted by restricting the search region to the external areas of the G-quadruplex, yielding active candidate **1** that was predicted to bind to the grooves of the c-*myc* G-quadruplex as indicated by NMR and molecular modeling studies. *In vitro* assays on a series of related compounds generated a brief structure-activity relationship for carbamide derivatives. To our knowledge, this is the first application of high-throughput virtual screening of a natural product database to identify c-*myc* G-quadruplex groove-binders. Computer-based hit-to-lead optimization is currently being carried out in order to generate further analogues for *in vitro* testing.

## Materials and Methods

### Materials

DNA oligomers were obtained from Tech Dragon Limited (Carlsbad, CA). The sequences for the oligomers were:

Pu27 = [5′-TGGGGAGGGTGGGGAGGGTGGGGAAGG-3′]

Pu24I = [5′-TGAGGGTGGIGAGGGTGGGGAAGG-3′]

Pu27_mut_ = [5′-TGGGGAGGGTGGAAAGGGTGGGGAAGG-3′]

HTS = [5′-AGGGTTAGGGTTAGGGTTAGGGT-3′]

Carbamide **1** and the other tested compounds were purchased from Analyticon Discovery GmbH (Postdam, Germany) natural product database. This database, containing over 20,000 natural product/natural product-like structures, is publicly available and can be accessed free of charge. *Taq* DNA polymerase was purchased from QIAGEN (Valencia, CA). Stock solution of **1** (10 mM) was made in dimethyl sulfoxide (DMSO). Further dilutions to working concentrations were made with double-distilled water.

### PCR Stop Assay

The polymerase stop assay was performed by using a modified protocol of the previously reported method [9]. The reactions (20 µL) were performed in 1× PCR buffer, containing each pair of oligomers (10 µM), deoxynucleotide triphosphate (0.16 mM), Taq polymerase (2.5 U), and increasing concentrations of the compound **1** (from 0–250 µM). The reaction mixtures were incubated in a thermocycler under the following cycling conditions: 94°C for 3 min followed by 23 cycles of 94°C for 30 s, 58°C for 30 s, and 72°C for 30 s. The amplified products were resolved on 15% polyacrylamide gel and visualized by ethidium bromide staining.

### Molecular Modeling

Molecular docking was performed by using the ICM-Pro 3.6-1d program (Molsoft) [19], [25]. According to the ICM method, the molecular system was described by using internal coordinates as variables. Energy calculations were based on the ECEPP/3 force field with a distance-dependent dielectric constant. The biased probability Monte Carlo (BPMC) minimization procedure was used for global energy optimization. The BPMC global-energy-optimization method consists of 1) a random conformation change of the free variables according to a predefined continuous probability distribution; 2) local-energy minimization of analytical differentiable terms; 3) calculation of the complete energy including nondifferentiable terms such as entropy and solvation energy; 4) acceptance or rejection of the total energy based on the Metropolis criterion and return to step (1). The binding between **1** and DNA was evaluated by binding energy, including grid energy, continuum electrostatic, and entropy terms. The initial model of loop isomer was built from the X-ray crystal structures of human intramolecular telomeric G quadruplex (PDB code: 1KF1) [7], according to a previously reported procedure [9], [10]. Briefly, the structure of human intramolecular telomeric G quadruplex was imported into Insight II package (Accelrys Inc., San Diego, CA), and necessary modifications were carried out including replacements and deletions of bases. Missing loop nucleotides were added using single-strand B-DNA geometry using the Biopolymer module. Potassium ions were placed between the G-tetrad planes to stabilize the tetrad structure. The initial models were then immersed in a box of TIP3P water molecules, and an appropriate number of sodium ions was added to neutralize the negative charge of the phosphate backbone. The molecular dynamics simulations were carried out in NAMD with VMD monitoring the process. The CHARMM force field parameter was assigned to every atom, and the Particle Mesh Ewald electrostatics was used to compute long-range electrostatic interactions. Hydrogen atoms were added and minimized by 3000 steps of conjugate gradient minimization. After 4000 steps of conjugate gradient minimization, two stages of molecular dynamics simulations were carried out at 300 K. In the first stage, only the loop area atoms were allowed to move, and this process involved a 20 ps equilibration and 100 ps simulations. The second stage involved unrestrained molecular dynamics simulations with 20 ps equilibration and 100 ps simulations at 300 K. Trajectories were recorded every 0.1 ps, and the most stable structure was extracted and further refined by 2500 steps of conjugate gradient minimization. In the docking analysis, the binding site was assigned to the groove regions of the DNA molecule. The ICM docking was performed to find the most favorable orientation. The resulting trajectories of the complex between **1** and G-quadruplex DNA were energy minimized, and the interaction energies were computed.

### ^1^H NMR Titration Experiments

The oligonucleotide Pu24I was annealed in buffer containing 150 mM KCl, 25 mM KH_2_PO_4_, 1 mM EDTA, pH 7.0 at 95°C for 10 min followed by slowly cooling to room temperature. ^1^H NMR experiments were performed with a Bruker AV600 spectrometer fitted with a cryoprobe. Typical acquisition conditions for a ^1^H NMR spectrum were 90° pulse length, 2.0 s relaxation delay, 32 K data points, 16 ppm spectrum width, 64–128 transients, and Pu24I in 90% H_2_O/10% D_2_O with 150 mM KCl, 25 mM KH_2_PO_4_, 1 mM EDTA, pH 7.0. Aliquots of a stock solution of **1** in DMSO-d6 were titrated directly to the DNA solution inside an NMR tube. Spectra were recorded at 298 K utilizing a standard jump-return pulse sequence for water suppression with a relaxation delay of 2.0 s.

#### Cell culture

Human hepatocarcinoma (HepG2) cells were maintained in minimum essential medium (MEM) supplemented with fetal bovine serum (10%), penicillin (100 U mL^−1^), streptomycin (100 µg mL^−1^) at 37°C under a humidified atmosphere with 5% CO_2_.

### Reverse Transcriptase-polymerase Chain Reaction (RT-PCR)

HepG2 cells were grown in six-well plates for 24 h. The cells were treated with the indicated concentrations of **1** for 16 h. Total RNA was extracted according to the manufacturer’s (Qiagen) instruction. Reverse transcription was carried out by incubating RNA (1 µg), random primers (100 ng) and dNTPs (0.5 mM) at 42°C for 5 min, followed by further incubation with 200 units of SuperScript II reverse transcriptase in 1×First-Strand buffer at 42°C for 30 min for reverse transcription and then at 95°C for 3 min to inactivate the enzyme. PCRs (containing cDNA (1 µg), dNTPs (10 mM), *Taq* polymerase (2.5 U), 10×PCR buffer and 10 µM of each primer) were performed for 22 cycles, consisting of 15 s at 95°C, 30 s at 58°C and 30 s at 72°C in a thermocycler, and the extension time was increased to 5 min during the last cycle. The amplified products were resolved on 1.3% agarose gel and visualized by ethidium bromide staining.

## Supporting Information

Figure S1 **NMR titration of 1 against the c-** ***myc*** **G-quadruplex Pu24I.** [Pu24I]/[**1**] = 1∶0 (lower panel) and 1∶2 (upper panel).
(DOCX)

Figure S2 **Overlay spectra showing G6 and G17 from three independent NMR titration experiments of 1 against the c-** ***myc*** **G-quadruplex Pu24I.** [Pu24I]/[**1**] = 1∶0 or 1∶2.
(DOCX)

Table S1 **Chemical shifts of G6 and G17 from three independent NMR titration experiments with statistical analysis.**
(DOCX)
