# Correction: Discovery of a Natural Product-Like c-*myc* G-Quadruplex DNA Groove-Binder by Molecular Docking

**DOI:** 10.1371/annotation/632f2b99-dd0b-443e-9269-1156d0e7521f

**Published:** 2012-10-25

**Authors:** Dik-Lung Ma, Daniel Shiu-Hin Chan, Wai-Chung Fu, Hong-Zhang He, Hui Yang, Siu-Cheong Yan, Chung-Hang Leung

There were errors in the Funding section. The correct funding information is as follows: This work is supported by Hong Kong Baptist University (FRG2/11-12/009), Environment and Conservation Fund (ECF Project 3/2010), Centre for Cancer and Inflammation Research, School of Chinese Medicine (CCIR-SCM, HKBU), the Health and Medical Research Fund (HMRF/11101212), the Research Grants Council (HKBU/201811), the University of Macau (SRG013-ICMS12-LCH, MYRG091(Y1-L2)-ICMS12-LCH and MYRG121(Y1-L2)-ICMS12-LCH), and Special Equipment Grant (SEG_PolyU01) of the University Grants Committee. The funders had no role in study design, data collection and analysis, decision to publish, or preparation of the manuscript.

