# Supplementary figures and images for: Discovery of a Natural Product-Like c-myc G-Quadruplex DNA Groove-Binder by Molecular Docking
Source: PLoS One. 2012 Aug 17;7(8):e43278. doi: 10.1371/journal.pone.0043278 (PMC3422278; doi:10.1371/journal.pone.0043278)

**
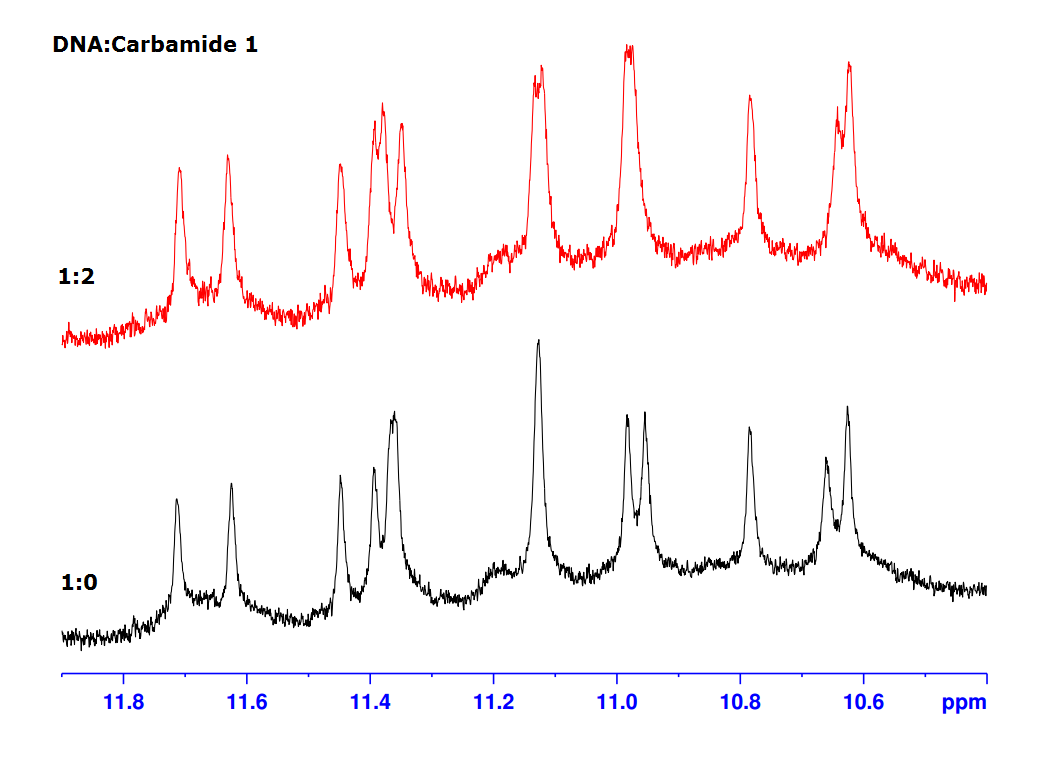
**

**Figure S1** NMR titration of **1** against the c-*myc* G-quadruplex Pu24I. [Pu24I]/[**1**] = 1:0 (lower panel) and 1:2 (upper panel).

Supplement: Figure S1 — NMR titration of 1 against the c- myc G-quadruplex Pu24I. [Pu24I]/[1] = 1∶0 (lower panel) and 1∶2 (upper panel). (DOCX) [file pone.0043278.s001.docx]
