# Supplementary material for: Discovery of a Natural Product-Like c-myc G-Quadruplex DNA Groove-Binder by Molecular Docking
Source: PLoS One. 2012 Aug 17;7(8):e43278. doi: 10.1371/journal.pone.0043278 (PMC3422278; doi:10.1371/journal.pone.0043278)
Supplement: Figure S2 — Overlay spectra showing G6 and G17 from three independent NMR titration experiments of 1 against the c- myc G-quadruplex Pu24I. [Pu24I]/[1] = 1∶0 or 1∶2. (DOCX) [file pone.0043278.s002.docx]

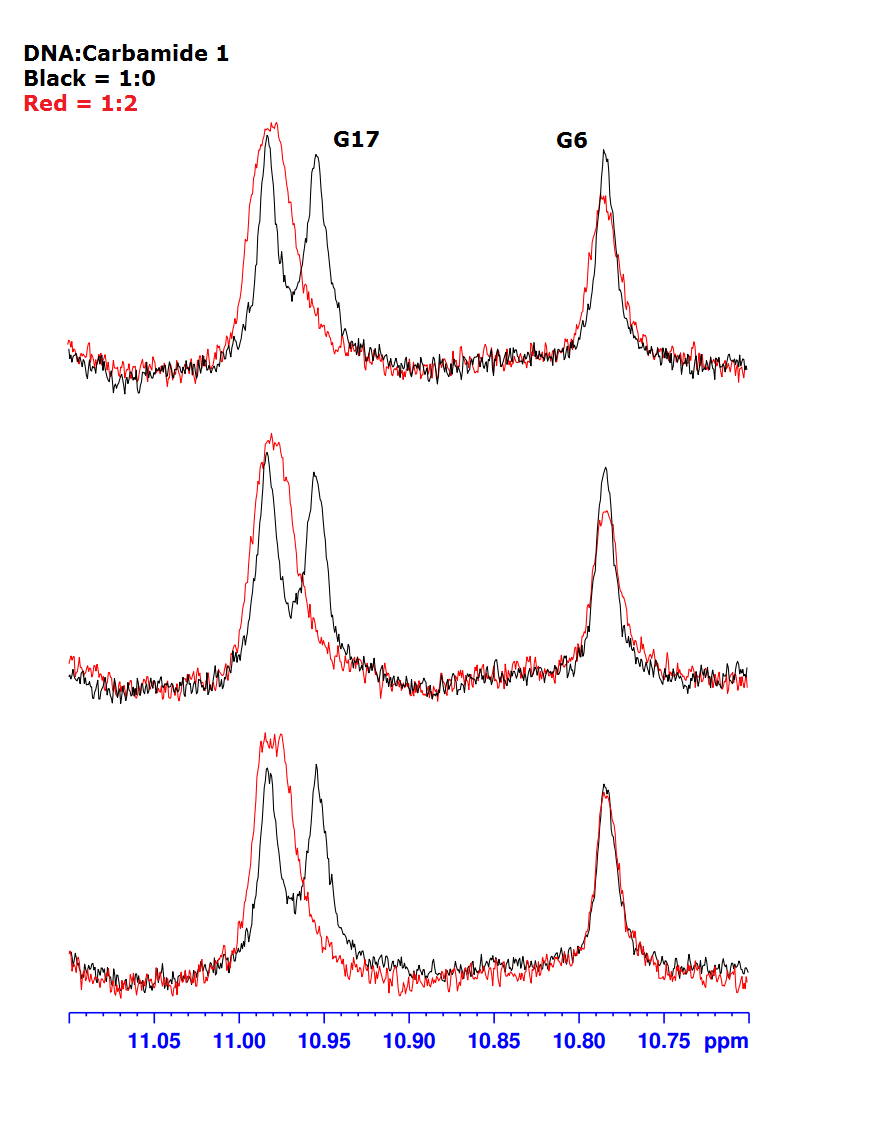
**Figure S2** Overlay spectra showing G6 and G17 from three independent NMR titration experiments of **1** against the c-*myc* G-quadruplex Pu24I. [Pu24I]/[**1**] = 1:0 or 1:2.
