# Supplementary material for: Discovery of a Natural Product-Like c-myc G-Quadruplex DNA Groove-Binder by Molecular Docking
Source: PLoS One. 2012 Aug 17;7(8):e43278. doi: 10.1371/journal.pone.0043278 (PMC3422278; doi:10.1371/journal.pone.0043278)
Supplement: Table S1 — Chemical shifts of G6 and G17 from three independent NMR titration experiments with statistical analysis. (DOCX) [file pone.0043278.s003.docx]

|  | Chemical shifts | | | | | |
| --- | --- | --- | --- | --- | --- | --- |
|  | G6 | | | G17 | | |
| Trials | 1 | 2 | 3 | 1 | 2 | 3 |
| Pu24I:Carbamide = 1:0 | 6472 Hz | 6471 Hz | 6471 Hz | 6574 Hz | 6574 Hz | 6573 Hz |
| Pu24I:Carbamide = 1:2 | 6471 Hz | 6471 Hz | 6471 Hz | 6586 Hz | 6588 Hz | 6588 Hz |
| Shift in Hz | 1 | 0 | 0 | 12 | 14 | 15 |
| Shift in ppm | 0.0016 | 0.0000 | 0.0000 | 0.020 | 0.023 | 0.025 |
|  |  |  |  |  |  |  |
| Average Shift in ppm | 0.00053 | | | 0.02267 | | |
| Standard Deviation in ppm | 0.00092 | | | 0.00252 | | |

**Table S1** Chemical shifts of G6 and G17 from three independent NMR titration experiments with statistical analysis.
